# A rare case of asymptomatic giant pulmonary hamartoma

**DOI:** 10.1186/s13000-024-01506-0

**Published:** 2024-06-22

**Authors:** Xiaoming Fan, Barry Breaux, Laura Leonards, Rusella Mirza

**Affiliations:** 1 Department of Pathology and Translational Pathobiology, Louisiana State University Health Shreveport, 1501 Kings Highway, Shreveport, LA 71103 USA; 2 Pathology Department, North Oaks Medical Center, North Oaks Health System, Hammond, LA USA; 3 North Oaks Imaging Associates, North Oaks Health System, Hammond, LA USA

**Keywords:** Pulmonary hamartoma, Chondromyxoid neoplasm, Malignant sarcomatous transformation

## Abstract

**Background:**

Pulmonary hamartomas are benign lung lesions. Histopathologically, pulmonary hamartoma is composed of varying amounts of mesenchymal elements, including chondroid tissue, mature adipose tissue, fibrous stroma, smooth muscle, and entrapped respiratory epithelium. Most pulmonary hamartoma cases are asymptomatic and found incidentally during imaging. They usually appear as well-circumscribed lesions with the largest dimension of less than 4 cm. Asymptomatic giant pulmonary hamartomas that more than 8 cm are rare.

**Case presentation:**

In the current case report, a 12.0 × 9.5 × 7.5 cm lung mass was incidentally noticed in a 59-year-old female during a heart disease workup. Grossly, the lesion was lobulated with pearly white to tan-white solid cut surface and small cystic areas. Microscopically, representative tumor sections demonstrate a chondromyxoid appearance with relatively hypocellular stroma and entrapped respiratory epithelium at the periphery. No significant atypia is noted. No mitosis is noted, and the proliferative index is very low (< 1%) per Ki-67 immunohistochemistry. Mature adipose tissue is easily identifiable in many areas. Histomorphology is consistent with pulmonary hamartoma. A sarcoma-targeted gene fusion panel was further applied to this case. Combined evaluation of microscopic examination and sarcoma-targeted gene fusion panel results excluded malignant sarcomatous transformation in this case. The mediastinal and hilar lymph nodes are histologically benign. After surgery, the patient had an uneventful postoperative period.

**Conclusions:**

Giant pulmonary hamartoma is rare; our case is an example of a huge hamartoma in an asymptomatic patient. The size of this tumor is concerning. Thus, careful and comprehensive examination of the lesion is required for the correct diagnosis and to rule out co-existent malignancy.

## Background

Pulmonary hamartomas are benign lung lesions. They usually occur in middle-aged or elderly adults, with the peak incidence in the sixth and seventh decade of life. Males are more frequently affected than females (approximately a 2:1 ratio) [1, 2]. Most pulmonary hamartomas are found in the lung parenchyma, with only a small portion in the endobronchial [1]. Histopathologically, pulmonary hamartoma is composed of varying amounts of mesenchymal elements, including chondroid tissue, mature adipose tissue, fibrous stroma, smooth muscle, and entrapped respiratory epithelium. Most pulmonary hamartoma cases are asymptomatic and found incidentally during imaging. They usually appear as well-circumscribed lesions with the largest dimension less than 4 cm [3, 4]. Giant pulmonary hamartomas larger than 8 cm are rare, with less than 25 cases reported in the English literature to date, and only 4 cases documented within the past 5 years. Typically, giant pulmonary hamartomas manifest with pulmonary-related symptoms like dyspnea, cough, chest pain, or hemoptysis [5]. Asymptomatic giant pulmonary hamartomas are even rarer, with only 6 cases reported thus far [5]. Here, we are presenting a giant pulmonary hamartoma (12.0 cm) case that was an incidental finding during a heart disease workup and without significant pulmonary symptoms.

## Case presentation

**Fig. 1:**
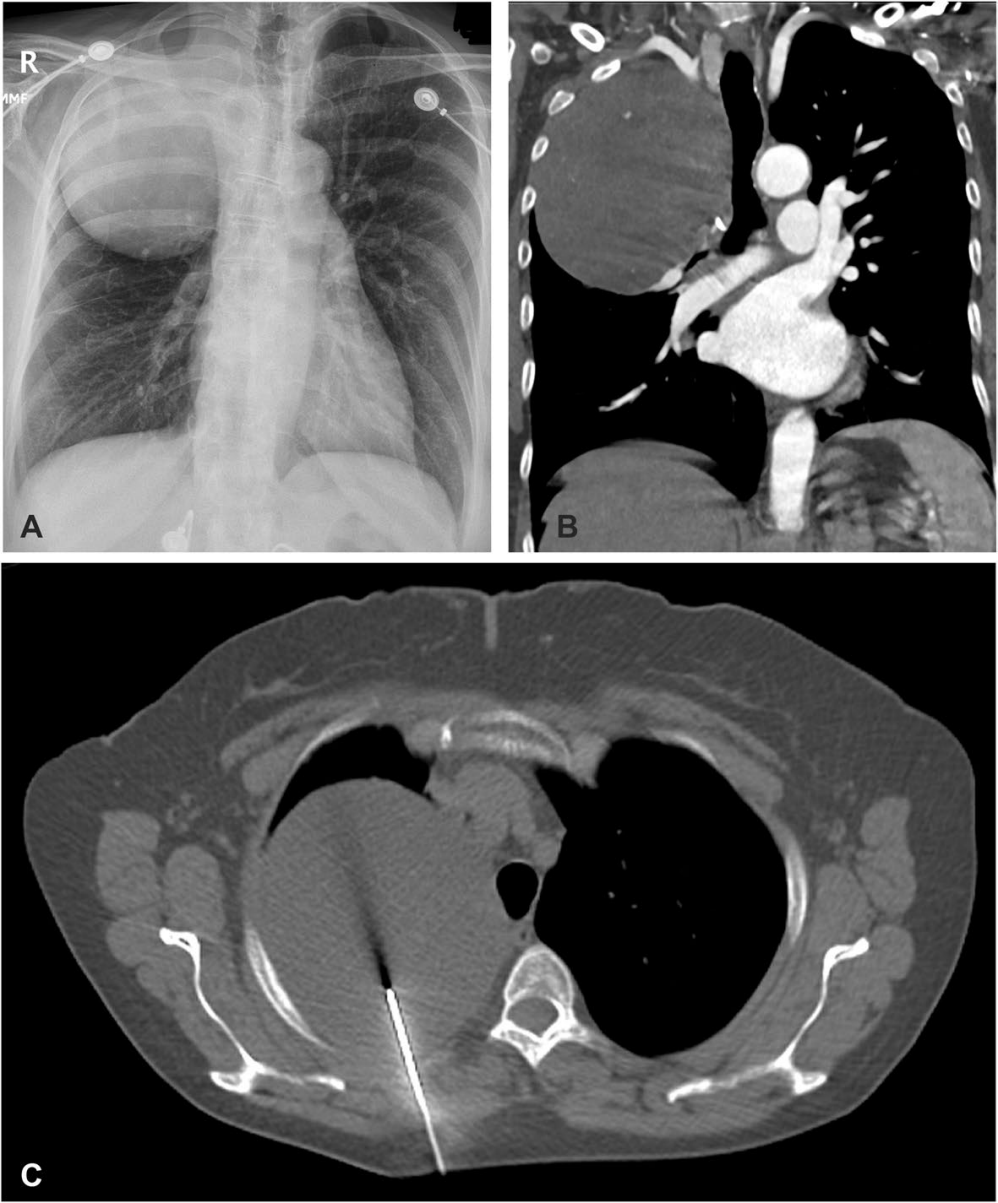
Radiology images of the pulmonary mass. Chest X-ray (**A**) and CT (**B** and **C**) demonstrate a well-circumscribed right upper lung mass that abuts the trachea but does not compress the airway. The lesion shows broad pleural abutment without invasion of the chest wall or ribs. The tiny hyperdense focus in the central upper portion of the mass represents calcium deposits (**B**)

A 59-year-old female presented to the emergency room with anxiety and hypertension due to heart problems. She has no history of malignancy or any other neoplasm. A right upper lobe lung mass was incidentally noticed in the chest radiograph. Chest further radiology evaluation demonstrated a well-circumscribed lesion in the upper right lobe of the lung, measuring approximately 11.8 × 10.9 × 10.2 cm. The mass abuts the trachea and shows broad pleural abutment without invasion of the chest wall or ribs or compressing the airway. The lesion had internal calcification and calcified granuloma (Fig. 1). A needle biopsy of the lesion demonstrated a chondromyxoid neoplasm.

**Fig. 2:**
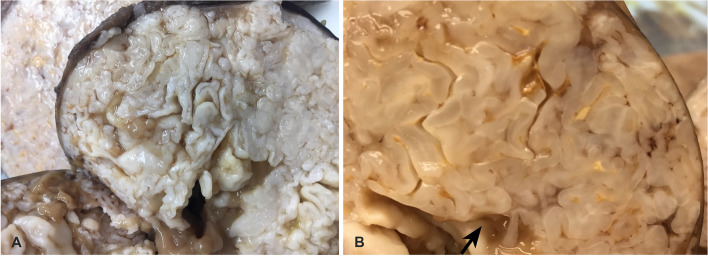
Gross appearance of the giant hamartoma. Sectioning of lesion demonstrates pearly white, lobulated (**A**), and chondromyxoid (**B**) cut surface with a small amount of mucoid material (arrow)

**Fig. 3:**
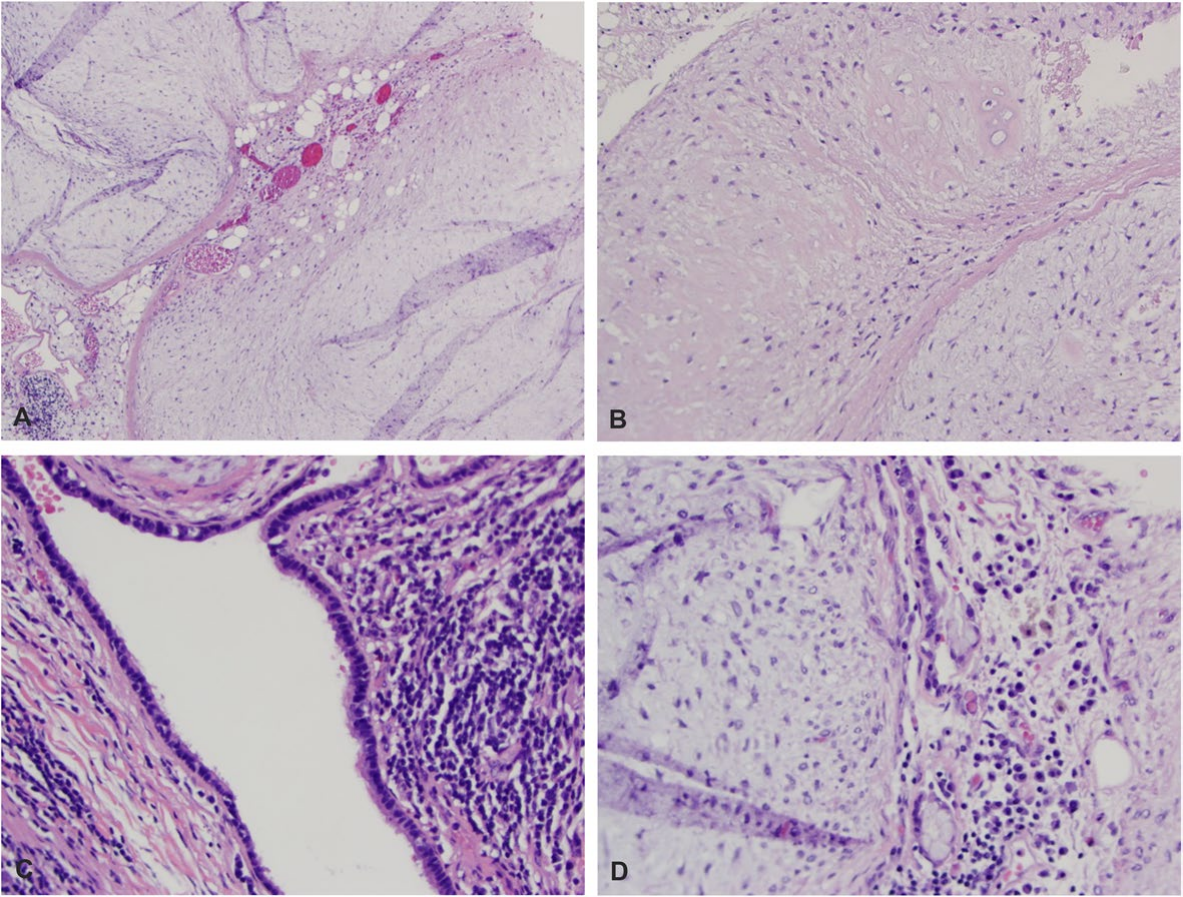
Histopathological features of the giant hamartoma. Myxoid hypocellular stroma with adipose tissue (**A**), chondroid differentiation (**B**), entrapped ciliated respiratory epithelium, and bronchial glands (**C**, **D**)

Partial lobectomy of the right upper lung and dissection of mediastinal and hilar lymph nodes were performed. Gross pathological examination of the tumor revealed a light pink to tan-white well-circumscribed mass measuring 12.0 × 9.5 × 7.5 cm. The cut surface of the lesion was predominantly lobulated, pearly white to tan-white solid with small cystic areas. Some areas with mucoid material were noted (Fig. 2). Microscopically, representative tumor sections demonstrate chondromyxoid tumor forming multiple lobules with relatively hypocellular stroma (Fig. 3). There are entrapped ciliated respiratory epithelium and mucin-producing-bronchial glands at the periphery in between lobules, highlighted by positive ae1/ae3 immunohistochemistry. Chondromyxoid stroma shows focal positivity with s100. No significant atypia is noted. No mitosis is noted, and the proliferative index is very low (< 1%) per Ki-67 immunohistochemistry. Mature adipose tissue is easily identifiable in many areas. Histomorphology was consistent with pulmonary hamartoma. Sarcoma Targeted Gene Fusion/Rearrangement Panel (Test ID: SARCP, Mayo Clinic Laboratory, Rochester, MN) was used to exclude malignant sarcomatous transformation in the current case. This panel evaluates 138 gene targets for the presence of somatic gene fusions observed in various sarcomas and also covers the gene fusions associated with pulmonary hamartoma. The mediastinal and hilar lymph nodes are benign. After surgery, the patient had an uneventful postoperative recovery. Subsequent clinical follow-ups at 2 months and 6 months, along with radiological follow-up at 4 months, revealed no abnormal changes.

## Discussion and conclusions

Pulmonary hamartomas are the most common benign neoplasms in adults [1]. The mean size of these hamartomas varies from 1.6 cm to 2.9 cm across different study series [1, 2, 4], with the largest size rarely exceeding 8.0 cm in those studies. To the best of our knowledge, there were fewer than 25 cases reported in the English literature to date with the largest size reported being 30.0 cm by Zong et al. [6] in 2014. Geramizadeh et al. [5] reviewed a series of giant pulmonary hamartoma (> 8.0 cm) case reports in 2019 and showed that although pulmonary hamartoma is typically an asymptomatic incidental finding, giant pulmonary hamartomas may present with pulmonary-related symptoms such as dyspnea, cough, chest pain, or hemoptysis [5, 7–12]. Only a few cases (~ 30%) were documented as asymptomatic or lacking pulmonary symptoms, like the current case. In the current case, the pulmonary hamartoma appeared as an asymptotic solid-cystic lesion with a size of 12.0 cm, making it a particularly unique instance.

Most pulmonary hamartomas are found in the lung parenchyma, with only a small portion in the endobronchial [1]. The components of these hamartomas can vary slightly, with different types of tissue being predominant [1]. In endobronchial pulmonary hamartomas, the predominant mesenchymal components include chondroid tissue (50% of cases), adipose tissue (33%), fibroblastic (8%), and osseous (8%). Conversely, in parenchymal pulmonary hamartomas, the primary components are chondroid tissue (80%), fibroblastic (12%), adipose tissue (5%), and osseous (3%). Particularly in parenchymal pulmonary hamartoma, fibroblasts may outgrow into alveolar walls, forming epithelial inclusions of type II pneumocytes or ciliated, non-ciliated, or mucus-producing bronchiolar cells. Chondromyxoid tissue was the predominant component in our case, with the mature adipose tissue following. Meanwhile, mucus-producing bronchiolar cells are significant, potentially contributing to the formation of the cystic structure in the current case. Other conditions, such as intrapulmonary teratoma or pleomorphic adenoma of the lung, may also demonstrate the combination of the significant amount of mucous glands and mesenchymal components. Pulmonary hamartoma can also be mistaken for pulmonary chondroma, which is part of Carney's triad, or it may appear as a component of Cowden syndrome. Carney’s triad, predominantly affecting young females, comprises synchronous or metachronous occurrences of gastric gastrointestinal stromal tumors (GIST), pulmonary chondroma, and extra-adrenal paraganglioma [13]. Pulmonary chondroma can be distinguished from pulmonary hamartoma by the absence of entrapped respiratory epithelium and secondary mesenchymal elements [14]. Cowden syndrome, on the other hand, is characterized by the presence of multiple hamartomas in various body regions, including the skin and internal organs, alongside distinctive mucocutaneous lesions and macrocephaly [15]. In the current case, from a clinical perspective, there's no record of prior neoplasms in the patient's history. Histologically, the typical solid-cystic architecture and classical predominant chondromyxoid tissue components, along with other secondary mesenchymal components in the pathological appearance, confirmed the diagnosis and ruled out those possibilities.

Previous studies have shown a high frequency of translocation t (3;12) in pulmonary hamartoma, resulting in HMGA2-LPP fusion transcripts [16]. The overexpression of HMGA2-LPP fusion transcripts may contribute to chondrogenesis and adipogenesis in pulmonary hamartoma [17]. In addition, rearrangements involving the RAD51L gene have also been reported in the literature [18]. In the Gene Fusion/Rearrangement Panel used in our current study, we investigated 2 RAD51L-associated gene fusions and 8 HMGA2-related gene fusions. Among these fusion transcripts, HMGA2-LPP fusion transcripts (fusing exon 3 of HMGA2 to exon 9 of LPP) and LPP-HMGA2 fusion transcripts (fusing exon 7 of LPP to exon 4 of HMGA2 variant and exon 8 of LPP to exon 4 of HMGA2 variant) have been reported to be associated with pulmonary hamartoma [19, 20]. However, we did not identify any of these reported gene fusions in the current case. This discrepancy may be attributed to the fact that not all pulmonary hamartomas harbor these gene rearrangements. In a serial study involving 30 cases [21], authors reported that rearrangements involving the HMGA2 gene and its surrounding area were observed in about 70% (21/30) of the cases. However, in another study involving 61 karyotypically normal pulmonary hamartomas, authors observed only 1 case harboring the HMGA2-LPP fusion transcript [22].

Although pulmonary hamartoma is generally considered a benign lesion, the coexistence of adenocarcinoma [23] or malignant transformation into leiomyosarcoma [24], liposarcoma [25], chondrosarcoma [26–29], and squamous cell carcinoma [30] have been reported. Most of the malignant transformed pulmonary hamartoma had a large size (> 8 cm). Therefore, careful investigation is recommended to rule out malignant transformation [29], especially in giant pulmonary hamartomas. In our case, given the giant nature of the tumor, in addition to careful microscopic examination, a Sarcoma Targeted Gene Fusion/Rearrangement Panel (Test ID: SARCP, Mayo Clinic Laboratory, Rochester, MN) was applied to rule out the malignant transformation. No reportable gene fusions informative for diagnosis, prognosis, or predictive response to therapy were identified. Based on the combined evaluation of the microscopy examination and the sarcoma gene fusion panel results, it appears that there are no alternate diagnoses other than pulmonary hamartoma.

Taken together, giant pulmonary hamartoma is rare, and our case is an example of a huge hamartoma in an asymptomatic patient. The size of this tumor is concerning. Thus, careful and comprehensive examination of the lesion is required for the correct diagnosis and to rule out co-existent malignancy.

## Data Availability

No datasets were generated or analysed during the current study.
